# PCL and DMSO_2_ Composites for Bio-Scaffold Materials

**DOI:** 10.3390/ma16062481

**Published:** 2023-03-21

**Authors:** Jae-Won Jang, Kyung-Eun Min, Cheolhee Kim, Chien Wern, Sung Yi

**Affiliations:** 1 Department of Mechanical and Material Engineering, Portland State University, Portland, OR 97201, USA; 2 Welding and Joining R&D Group, Korea Institute of Industrial Technology, 156, Getbeol-ro, Yeonsu-gu, Incheon 21999, Republic of Korea

**Keywords:** biomaterial, scaffold, additive manufacturing, material property, property tailoring

## Abstract

Polycaprolactone (PCL) has been one of the most popular biomaterials in tissue engineering due to its relatively low melting temperature, excellent thermal stability, and cost-effectiveness. However, its low cell attraction, low elastic modulus, and long-term degradation time have limited its application in a wide range of scaffold studies. Dimethyl sulfone (DMSO_2_) is a stable and non-hazardous organosulfur compound with low viscosity and high surface tension. PCL and DMSO_2_ composites may overcome the limitations of PCL as a biomaterial and tailor the properties of biocomposites. In this study, PCL and DMSO_2_ composites were investigated as a new bio-scaffold material to increase hydrophilicity and mechanical properties and tailor degradation properties in vitro. PCL and DMSO_2_ were physically mixed with 10, 20, and 30 wt% of DMSO_2_ to evaluate thermal, hydrophilicity, mechanical, and degradation properties of the composites. The water contact angle of the composites for hydrophilicity decreased by 15.5% compared to pure PCL. The experimental results showed that the mechanical and degradation properties of PCL and DMSO_2_ were better than those of pure PCL, and the properties can be tuned by regulating DMSO2 concentration in the PCL matrix. The elastic modulus of the composite with 30 wt% of DMSO2 showed 532 MPa, and its degradation time was 18 times faster than that of PCL.

## 1. Introduction

The goal of bone tissue engineering (BTE) is to regenerate or replace damaged bone [1,2,3]. Bone has a self-healing ability, but in critical-sized damage cases, bone tissue cannot be healed itself completely [4,5]. Therefore, external treatment is required in the latter case including autografts and allografts [6,7,8]. Although allograft is a main practice for repairing damaged bone tissues, a shortage of donors has still remained [9,10,11]. According to Health Resources & Services Administration (HRSA) data, over one hundred thousand people are on the national transplant waiting list, but only 40,000 transplants were performed in 2021 [12].

Porous scaffold, which mimics extracellular matrix (ECM), is a crucial technology for bone tissue engineering for cell attachment, proliferation, and supporting the human body [13]. ECM is a three-dimensional structure providing structural and biochemical support to surrounding cells in vivo [14]. Scaffold should have biocompatibility, biodegradability, and proper mechanical properties [15,16,17]. Moreover, geometry properties, fabrication methods, and biomaterials are also considered for scaffold manufacturing [18,19,20].

Scaffold materials can be divided into four groups: natural and synthetic polymers, bio-ceramics, metallic materials, and composites [21,22]. The synthetic polymer group is the most interesting material group because this group showed tunable mechanical and biodegradable properties as well as cost effect [23,24,25]. Polycaprolactone (PCL) is one of the famous synthetic bio-degradable polymers along with polylactic acid (PLA) and polyglycolide (PGA) [26]. PCL is already approved by Food and Drug Administration (FDA) as a biomaterial [27], and it has a relatively low melting temperature and excellent thermal stability [28] as well as a cheap price [29]. PCL, however, has a very slow degradation time, and low cell attractive property because of its hydrophobic [30]. Moreover, relatively high viscosity of PCL needs high pressure when it is printed and it makes it hard to print accurately [31]. In spite of these disadvantages, PCL is widely used in various tissue engineering applications due to its attractive properties as mentioned above. Many of PCL-based composites are reported to overcome PCL’s disadvantages [26].

Dimethyl sulfone (DMSO_2_), known as Methylsulfonylmethane (MSM), is an organosulfur compound occurred naturally [32]. DMSO_2_ is well known as an extremely stable and non-hazardous material, and it uses its unique properties to alter physiology at both the cellular and tissue levels [33]. DMSO_2_ can also act as a carrier or co-transporter for other therapeutic agents, further expanding its applications [34]. Moreover, DMSO_2_ can play a role in cartilage preservation [35]. There has long been a belief that cartilage degradation is one of the main reasons of osteoarthritis [36]. Articular cartilage is presented by a dense ECM and extracts nutrients from the adjacent synovial fluid with little to no blood [37]. In vitro studies indicate that DMSO_2_ can protect cartilage [38], and it possibly normalizes hypoxia-driven alterations to cellular metabolism [39]. DMSO_2_ has a low viscosity [40] that affects printing accuracy and high surface tension [41] which can improve hydrophilicity then can increase cell attractive property as a composite material in the biomaterial matrix [42].

A number of studies have attempted to improve the biomaterial properties of PCL-based scaffolds by conducting research on PCL-based composites [43,44,45,46]. PCL showed the slowest degradation time among polyester group polymers [15,47]. Fully degraded time of PCL was noted for about 2–3 years. PCL and 20 wt% of tricalcium phosphate (TCP) were fully degraded after 54 h even though the PCL scaffold took 6 weeks to entirely degrade in the accelerated degradation studies using 5 M sodium hydroxide (NaOH). Fifteen wt% of nano-hydroxyapatite (HA) decreased water contact angle of PCL from 112.98–79.50° [48]. Cellulose nanocomposites (CNC) were used to increase the elastic modulus of PCL-based composites [49]. However, there have not been enough studies of PCL-based composites to apply bone tissue engineering.

In this study, PCL and DMSO_2_ composites were investigated as a new bio-scaffold material to increase the hydrophilicity and mechanical properties, and to tailor the degradation property in vitro. In detail, the tailored properties of PCL by mixing DMSO_2_ with 10, 20, and 30 wt% of DMSO_2_ were studied. Material properties including thermal, hydrophilicity, mechanical, and degradation properties of composites were measured. Hydrophilicity and modulus of composites were increased with the concentration of DMSO_2_ in the PCL matrix, and degradation time was accelerated 1.2–18 times more than the pure PCL by DMSO_2_ ratio. By regulating DMSO_2_ concentrations, the properties of composites can be tailored to the specific situation.

## 2. Experiments

### 2.1. Materials

In this study, polycaprolactone (PCL) and PCL based composites with dimethyl sulfone (DMSO_2_) were used for hydrophilicity, mechanical properties, and degradation time. Powder type PCL with a molecular weight 50,000 was provided from Polysciences (Warrington, PA, United States), and powder type DMSO_2_ was provided from Bergstrom Nutrition (Vancouver, WA, United States). Both material properties provided by the manufacturer are shown in Table 1. In the pre-test of a composites, over 40 wt% of DMSO_2_ were either too hard or brittle, resulting in them unsuitable for experiments. Composites were physically mixed with DMSO_2_ concentrations of 10, 20, and 30 wt% using an electric milling machine (YUESUO, Zhengzhou, China). All materials were dried at 45 °C under vacuum condition for one day before mixing. In this paper, the composites were denoted by PCL/D10, PCL/D20, and PCL/D30 according to the DMSO_2_ weight percent.

### 2.2. Melting Temperature

The specimens for hydrophilicity, mechanical properties, and degradation test were fabricated by mold casting and additive manufacturing. A dynamic differential scanning calorimetry (DSC) method was performed to set a melting and a 3D printing temperature with heating and cooling rate of 5 °C/min. The temperature range of the test was 30–150 °C. The equipment was STA 8000 (PerkinElmer, Waltham, MA, United States) having a heat-flux type DSC with balance resolution of 0.2 ug and the temperature accuracy of ±0.5 °C from ambient to 1000 °C. The heating rate was determined by considering the 3D printer heating speed and the cooling rate was set the same as the heating rate. The melting temperature was set at the onset temperature under endothermic process [50,51].

### 2.3. Hydrophilicity

**Figure 1:**
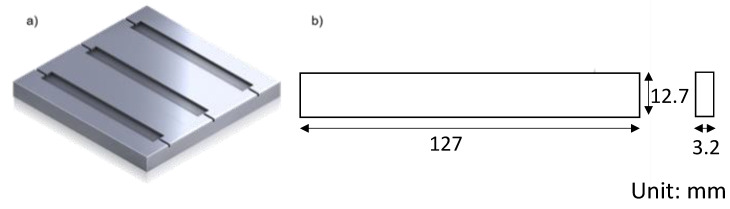
Mold design (**a**) and specimen sizes (**b**) for the mechanical test.

Specimens for hydrophilicity were fabricated by mold casting, and the target specimen size was 12.7 mm × 25.4 mm × 3.2 mm. The mold was designed according to the ASTM D790 standard [52] for the three-point bending test having 3 cavities of 127 mm × 25.4 mm × 3.2 mm (Figure 1). The solid material of pure PCL and mixed PCL and DMSO_2_ composites were put into the mold, and the mold was placed on a hot plate heated 120 °C. The mold with fully melted materials was cooled at room temperature for 2 h, and the top surface of melted materials was flattened by a metal plate having a smooth surface during solidification. Casted bar was cut into 5 pieces for the test.

Hydrophilicity can be evaluated using contact angle by the sessile drop method according to the ASTM D7334 standard [53]. A common test liquid of hydrophilicity is distilled (DI) water. In this test, DI water and phosphate-buffered saline (PBS) solution were used as a test liquid. The test liquid of 10 µL was dropped to the specimen and the angle between the test liquid and the specimen was measured at the endpoints of the left and right. The test was performed 5 times and the contact angle was denoted by an average value.

### 2.4. Mechanical Test and Failure Analysis

Mechanical test specimens were fabricated by mold casting. The mold was the same used in the hydrophilicity test. The test specimen sizes were 12.7 mm × 127 mm × 3.2 mm. The specimen manufacturing process was the same as the hydrophilicity test except for the cutting process.

Elastic modulus and 0.2% offset yield strength were measured by using the three-point bending test to evaluate the mechanical properties of the specimens. A bench-mounted universal testing machine of model 5ST (Tinius Olsen, Redhill, United Kingdom) was used for the test. The equipment has a maximum force of 5000 N with 0.2% load measuring accuracy of the reading 0.2–100% of the load cell capacity. The test was performed to the strain limit of 5%, and the test ended when the deflection of midspan of the materials reached to 6.8 mm. The rate of crosshead motion and the end condition of the test were calculated according to the ASTM D790 standard [52]. The test was performed using 5 samples, and the maximum and the minimum value were excepted when the mechanical properties were calculated.

The stress and the strain were calculated by

(1) $$\sigma =\frac{3PL}{2bd^{2}}$$

(2) $$\varepsilon =\frac{3Dd}{L^{2}}$$

where *σ* and *ε* are stress in the outer fibers at midpoint and strain in the outer surface, respectively. *P* is the load at a given point on the load-deflection curve, *L* is a support span, *b* is the width of tested beam, *d* is a thickness of tested beam and *D* is the maximum deflection of the center. Modulus in elasticity were obtained by load-deflection curve from above equations. Modulus in elasticity was calculated by

(3) $$E=\frac{L^{3}m}{3bd^{3}}$$

where *E* is modulus in elasticity (MPa), and *m* is the gradient of the tangent to the initial straight-line portion of the load-deflection curve (N/mm). 

In order to investigate the surface of the composites and the fracture surface after the test, a scanning electron microscope (SEM) analysis was performed. SNE-4500M Plus (SEC Co., Ltd., Suwon, Republic of Korea) was used. The surfaces of composites were coated with Au for 4 min using an ion sputter coater (MCM-100P, SEC Co., Ltd., Republic of Korea) before SEM analysis.

### 2.5. Degradation Test

**Figure 2:**
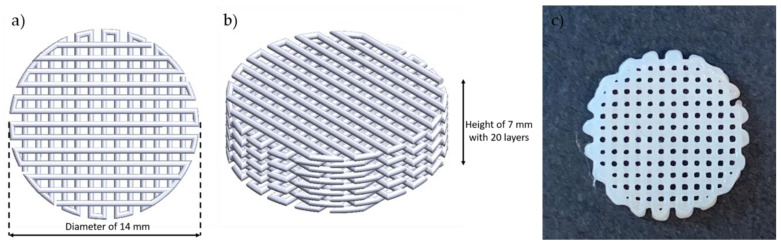
Scaffold design: (**a**) top view of designed scaffold, (**b**) isometric view of designed scaffold, and (**c**) top view of printed scaffold.

Degradation test specimens were fabricated by the material extrusion process using Biobot (current model: Allevi2, Allevi, Inc., Philadelphia, PA, United States), a desktop pneumatic Extrusion 3D bioprinter. This equipment can control pressure and temperature with ranges of 6.895–827.371 kPa and from room temperature to 160 °C, respectively. The specimen shape was a cylindrical scaffold with a diameter of 14 mm and 20 layered, and each layer has a 360 µm (Figure 2). The target porosity of the scaffold was 55–58% under the above printing conditions. The porosity of the scaffold was measured by the printed scaffold weight. Original scaffold structure was designed using SOLIDWORKS 2021 (Dassault Systèmes, Vélizy-Villacoublay, France), and then sliced using Slic3r software version 1.2.9. To achieve the same surface area for all specimens, printing temperature and pressure were varied from 120 °C to 130 °C and from 30 psi to 70 psi, individually, but the printing speed and the nozzle inner diameter were fixed at 0.8 mm/s and 450 µm, respectively.

The degradation test of the scaffold was performed in an environment of PBS solution at 37 °C. Scaffolds fully soaked into a container containing PBS solution and the containers were stored in the vacuum oven for 9 weeks with interval 3 weeks. Every 3 weeks during the whole period of the test, the mass of the scaffolds was measured using a digital milligram scale (GEMINI-20, Cumming, GA, United States) in 1 mg increments. The degradation rate was evaluated by mass loss percent. All scaffold was cleaned and dried before measuring.

## 3. Results

### 3.1. Melting Temperature

**Figure 3:**
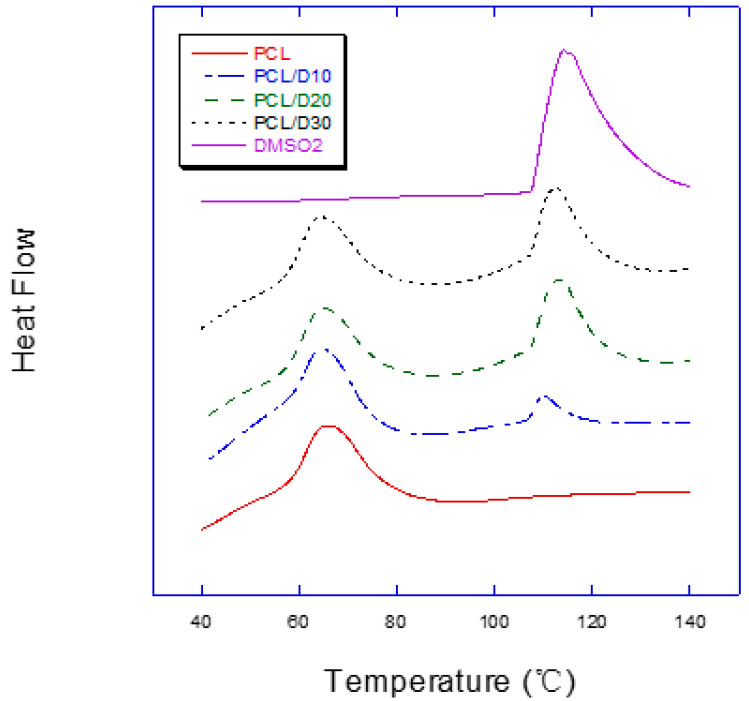
Dynamic DSC curves of PCL and DMSO_2_ composites.

In the DSC test result, the composites showed separated two peaks during the endothermic process while pure PCL and DMSO_2_ showed only one peak (Figure 3). The melting temperatures of PCL in the composites ranged 55.99–56.73 °C, and it of DMSO_2_ ranged 106.36–107.48 °C (Table 2). The temperature for specimen preparation was set as higher about 10%, 120 °C, because the specimen preparation process is not a closed system to be different from the DSC test condition.

### 3.2. Hydrophilicity of Composites

**Figure 4:**
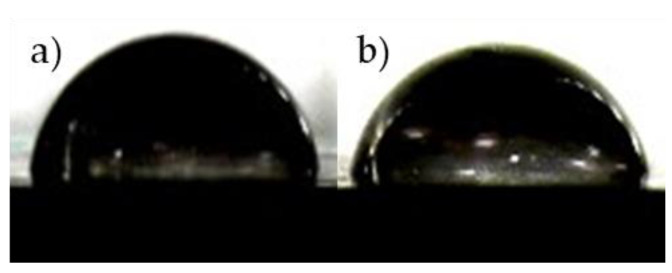
Water contact angles of PCL and DMSO_2_ composites: (**a**) pure PCL and (**b**) PCL/D30.

**Figure 5:**
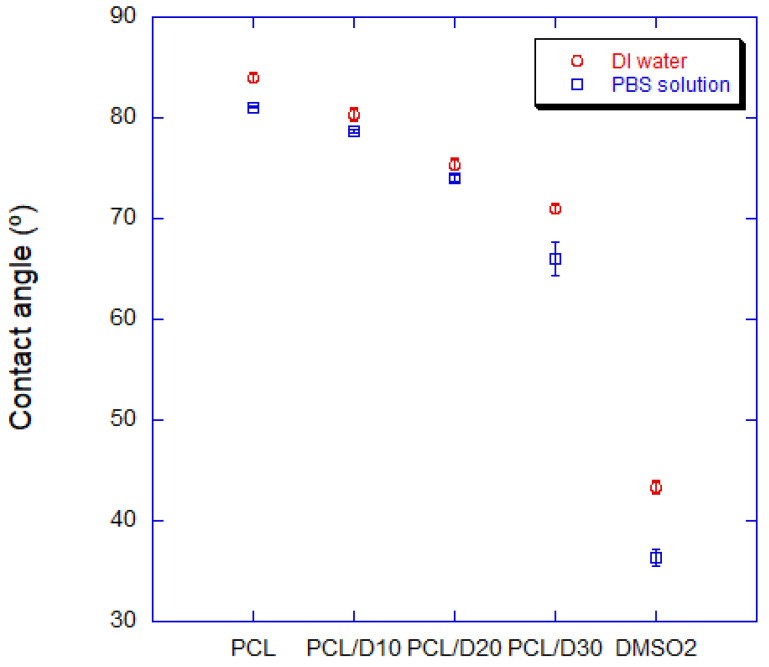
Contact angle of PCL and PCL/DMSO_2_ composites with DI water and PBS solution.

The hydrophilicity of PCL and DMSO_2_ composites was better than it of pure PCL. The water contact angle (WCA) of PCL and DMSO_2_ composites showed a decreasing angle value of 4.4, 10.2, and 15.5% compared to it of pure PCL according to the DMSO_2_ concentration interval of 10 wt%, respectively (Figure 4). The WCA of PCL was 83.94°, and it of DMSO_2_ was 43.35°, and PCL and DMSO_2_ composites showed the WCA of 80.28°, 75.37°, and 70.96°, individually. This tendency was similarly observed in the PBS solution. The contact angle of PCL and DMSO_2_ composites presented 78.63°, 74.05°, and 66.04° with 10, 20, and 30 wt% of DMSO_2_ ratio, individually. All materials showed low contact angle in PBS solution (Figure 5).

### 3.3. Mechanical Properties

**Figure 6:**
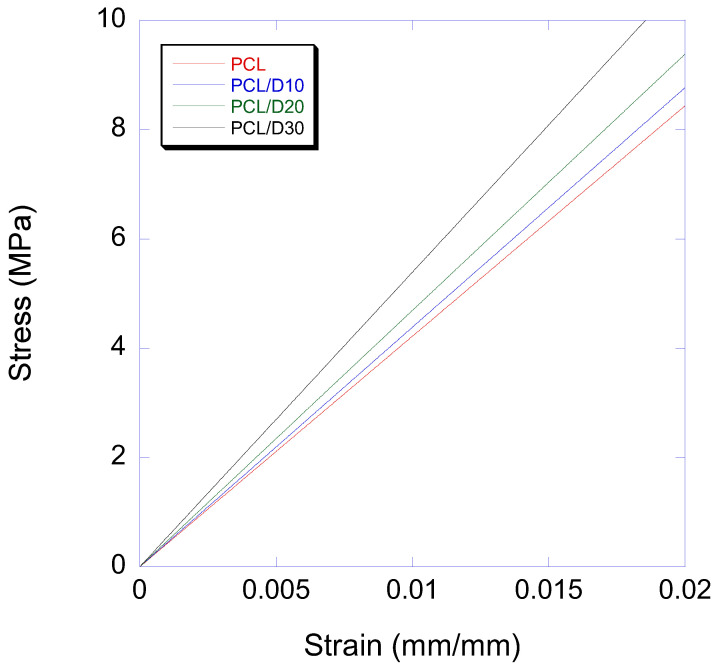
Stress-Strain curve of PCL and PCL/DMSO_2_ composites in the elastic region.

The elastic modulus of PCL and DMSO_2_ composites showed a linear relationship with the DMSO_2_ ratio with values of 440, 469, and 532 MPa at 10, 20, and 30 wt% of DMSO_2_ concentration in composites (Table 3), individually. These results indicated improvement rates of 3.8, 10.6, and 25.5% at each composite compared to pure PCL. The stress-strain curves in the elastic region were shown in Figure 6. It is clear that the addition of DMSO_2_ into PCL resulting in a positive change in the elastic modulus. The composites of PCL and DMSO_2_ can be considered as particle reinforced polymer matrix composites. Many researchers studied on the mechanical properties of particle reinforced polymer matrix composites [54,55,56,57]. Mechanical properties can be accordingly enhanced by adding micro- or nano-sized particles because most rigid particles have a higher stiffness than natural or synthetic polymer matrices [58,59].

In contrast to the positive relationship in elastic modulus between PCL and DMSO_2_, 0.2% offset yield strength of PCL and DMSO_2_ composites were decreased with increasing DMSO_2_ ratio. Strength including yield strength heavily depends on the stress transfer between polymer matrix and added particle, and well-bonded polymer matrix and particle can transfer enforced stress through the interface from polymer to the particle [60]. This process can clearly improve strength. In poor interface adhesion between polymer and particle, declined strength, however, could appear in composites, and this phenomenon occurs in micro-particle more frequently than nano-particle [61]. To observe interface adhesion, scanning electron microscope (SEM) analysis was conducted.

**Figure 7:**
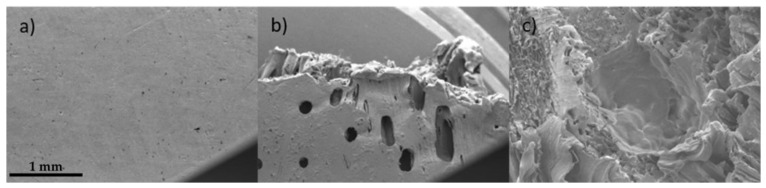
SEM images of PCL and DMSO_2_ composite with magnification ×50 (**a**) surface before mechanical test, (**b**) side view after mechanical test, and (**c**) top view after mechanical test.

The PCL and DMSO_2_ composites showed a clear surface without any defects (Figure 7). DMSO_2_ particles were pulled out from the PCL matrix due to low interface adhesion during the mechanical test, and stress is concentrated on the void. Stress concentrated void extended, and the fracture happened, finally. The evidence of pulling out of DMSO_2_ from PCL can be seen on the fracture surface.

### 3.4. Degradation Property In Vitro

**Figure 8:**
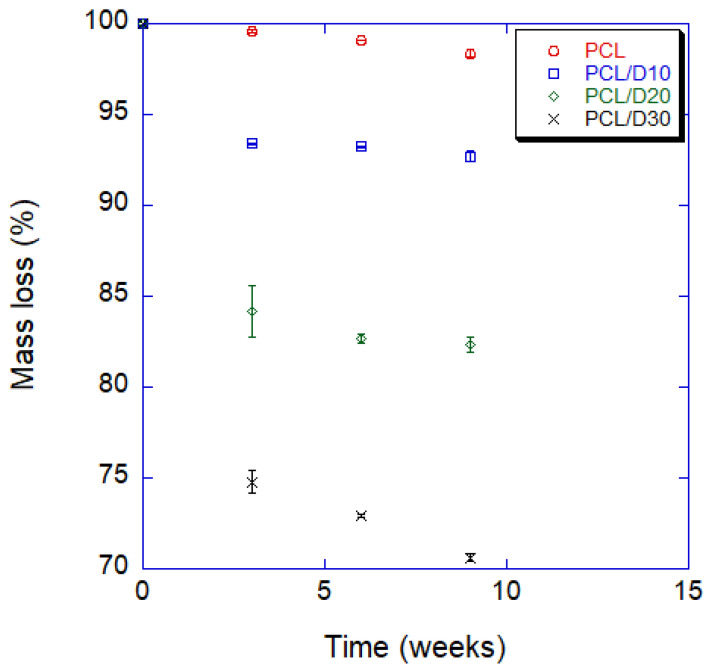
Mass loss for 9 weeks with interval 3 weeks of PCL and PCL/DMSO_2_ composites.

Three samples were tested for each composition, and mean values and standard deviations were proposed. After 9 weeks, the mass of pure PCL decreased a total of 1.62%, and it almost linearly decreased by 0.61% every 3 weeks on average. On the other hand, the mass loss of PCL and DMSO_2_ composites noticeably increased compared to pure PCL. The final mass losses of composites were totals of 7.29, 17.67, and 29.40% with 10, 20, and 30 wt% of DMSO_2_ concentration, respectively, and the mass loss was apparently faster in the first 3 weeks; after that, the mass loss rate gradually decreased until the end of the 9 weeks. Degradation rate by measuring mass loss was shown in Figure 8 and Table 4.

## 4. Discussion

The wettability of a polymer can be affected by its physical properties, particularly surface free energy. Therefore, it is possible to obtain PCL with varying molecular weights, resulting in different surface free energy and water contact angle [62,63]. In this study, PCL with 50,000 molecular weight was tested, and the literature data [64] used PCL with 80,000 molecular weight. This is the reason why the water contact angles of the literature data and the current study are different. High contact angle of pure PCL is caused by low surface energy which interrupts initial cell adhesion, leading to limit cell to cell or cell to matrix interactions [30,65]. DMSO_2_ can play a role reducing the liquid contact angle due to high surface energy and improving the process of cell attachment by adding to PCL matrix. Adding the DMSO_2_ in the PCL on hydrophilicity is better than other PCL-based composites. Moreover, the result of PCL/DMSO_2_ 30 wt% composite indicates the WCA of the composite can reach metallic biomaterial level such as titanium, although polymers generally show higher contact angle compared to that of ceramics and metals due to the surface energy difference (Table 5).

The contact angle of bio composite material can be calculated by young’s equation [77] and rule of mixture. Young’s equation relating surface energy of a solid, surface tension of a liquid and the interfacial tension between the liquid and solid is related to the contact angle as follows,

(4) $$\sigma_{s}=\sigma_{sl}+\sigma_{l}\cos\theta$$

where, $\sigma_{s}$ is surface energy of a solid, $\sigma_{sl}$ is the interfacial tension between the liquid and solid, $\sigma_{l}$ is surface tension of a liquid, and θ is the contact angle. Therefore, both the surface energy of a solid and the contact angle are closely related. Fowkes [78] proposed a simple equation to deal with the $\sigma_{sl}$ as function of $\sigma_{s}$ and $\sigma_{l}$ in the following way,

(5) $$\sigma_{sl}=\sigma_{s}+\sigma_{l}-2\sqrt{\sigma_{s}\sigma_{l}}$$

**Figure 9:**
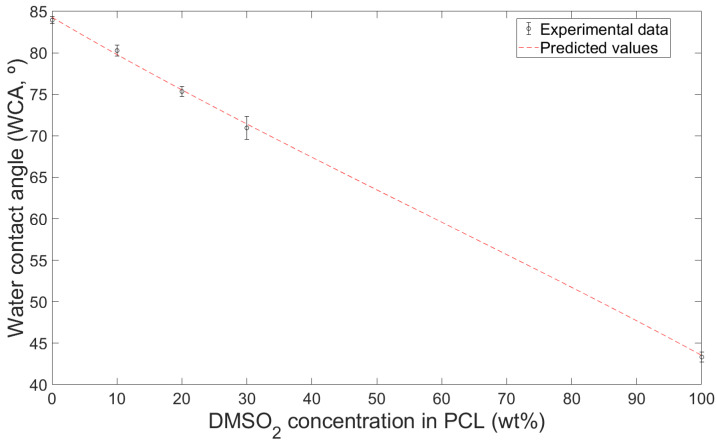
Contact angle with DI water of experiment value and predicted curve.

Combining Equations (4) and (5) to eliminate the term of $\sigma_{sl}$ and applying a general rule of mixtures, the contact angle can be predicted like Equation (6).

(6) $$\theta =\cos^{-1}\left(-1+2\sqrt{\frac{\sigma_{PCL}(1-f)+\sigma_{DMSO_{2}}f}{\sigma_{l}}}\right)$$

where $\sigma_{PCL}$ and $\sigma_{DMSO_{2}}$ are solid surface energy of PCL and DMSO_2_, respectively, and f is the fraction of DMSO_2_ in PCL and DMSO_2_ composites. Experiment contact angle value and predicted curve with DI water were shown in Figure 9. Coefficient of determination (R^2^) of predicted curve was 0.9116.

Hydrophilicity of biomaterial can be enhanced by surface treatment including etching, blasting, passivation, and plasma as well as blending other materials [79,80]. Strnad et al. [79] reported the surface treatment effects of acid etching, sandblasting, passivation, and their combinations on the titanium-based biomaterials. Long acid etching times affected a positive effect on hydrophilicity, but sandblasting did not influence significantly. Xu et al. [80] noted plasma surface treatment with time. After 150 min, the contact angle showed almost half the value compared with the initial contact angle. The hydrophilicity of PCL and DMSO_2_ composites might be more improved by surface treatment such as plasma treatment without changing DMSO_2_ concentration.

The elastic modulus of the single biomaterial, such as biopolymer, can be improved by fabricating composite material (Table 6), and the modulus is highly affected by test specimen design and additive materials [13,18]. PCL has a wide range of molecular weight, and thus the range of mechanical properties is also varied [62,81,82]. Perstorp (Malmö, Sweden) announced bulk PCL with a molecular weight of 50,000 had an elastic modulus of 470 MPa and 0.2% offset yield strength of 17.5 MPa [83]. However, PCL shows relatively low modulus compared to other synthetic biopolymers such as PLA and PGA [84]. Doyle et al. [85] studied the mechanical properties of PCL and nano-hydroxyapatite(nHA) blends with cylindrical disk specimens (7 mm diameter, 2 mm height) in the compressive test. The nHA showed negative effect in the low nHA concentration (10%), but in the high nHA concentration (30%) indicated higher modulus than PCL. Germiniani et al. [86] published about PCL and cellulose nanocrystals (CNC) composites. The mechanical test was performed according to ASTM D882-10. The modulus of composites was increased with CNC concentration, but in the CNC of 5%, the modulus was decreased. However, the modulus of PCL and DMSO_2_ composites was increased regardless of DMSO_2_ concentration unlike other additives.

DMSO_2_ is an organic sulfur, and the efficient breakdown of sulfide ions allows for further degradation of organic pollutants at an acidic pH, resulting in a highly effective treatment process [89]. This study showed that the degradation time of PCL and DMSO_2_ composite can tailor according to the DMSO_2_ ratio. A tailorable degradation time of the biomaterial can expand the use of the biomaterial because the human tissues have different regeneration times depending on the tissue and the extent of the wound [90,91]. In this study, the degradation time of the composites is reduced up to 18 times with DMSO_2_ of 30% after 9 weeks. The degradation time can be tailored more precisely by controlling the content of DMSO_2_ in the composites.

## 5. Conclusions

The purpose of this study was to develop a new bio-scaffold material using PCL and DMSO_2_ composites, and to investigate the hydrophilicity, mechanical, and degradation properties of the resulting materials. The experimental results showed that the PCL and DMSO_2_ composites had improved hydrophilicity and mechanical properties compared to pure PCL, and their degradation properties were tunable by regulating the concentration of DMSO_2_ in the PCL matrix.

1. The water contact angle decreased by 4.4%, 10.2%, and 15.5% with 10%, 20%, and 30 wt%, respectively, while the contact angle with PBS solution decreases 3.0%, 8.7%, and 18.5% with those. The water contact angle of the composites can be predicted using the surface tension of each material.
2. Adding DMSO_2_ to the PCL matrix increased the elastic modulus with increasing DMSO_2_ concentration rate. However, the 0.2% offset yield strength decreased with increasing DMSO_2_ ratio due to poor interfacial adhesion between PCL and DMSO_2_, which occurred more frequently with micro-sized particles than with nano-sized particles. The addition of extra additives, such as a binder, can be used to improve the yield strengths of the composites. The degradation rate should be regulated for specific conditions.
3. The degradation time of the composite with 30 wt% of DMSO_2_ was 18 times faster than that of pure PCL in a 9 week test. PCL and DMSO_2_ composites can tailor the degraded rate with DMSO_2_ ratio, and a wide range of degradation time can increase the selection for applications.

According to the above results, DMSO_2_ can play a role of increasing hydrophilicity, elastic modulus, and decreasing degradation time in the composites. Newly developed PCL/DMSO_2_ composites can be used as a bio-scaffold material in tissue engineering field.

## Figures and Tables

**Table 1:** Material properties of PCL and DMSO_2_ provided by the manufacturer.

Material	Appearance	Molecular Weight (g/mol)	Density (kg/m^3^)	Flash Point (°C)	Surface Tension (mN/m)	Viscosity (mPa·s)
PCL	Powder	50,000		275		
DMSO_2_	Powder	94.13	1450	143	60.15	1.14

**Table 2:** Melting temperature measured by DSC curves.

Material	Melting Temperature (°C)			
	Peak 1		Peak 2	
	Mean	SD	Mean	SD
PCL	56.59	0.16		
PCL/D10	55.39	0.52	106.82	0.30
PCL/D20	56.40	0.20	106.67	0.31
PCL/D30	56.27	0.07	106.73	0.31
DMSO_2_			106.85	0.46

**Table 3:** Elastic modulus and 0.2% offset yield strength of PCL and PCL/DMSO_2_ composites.

	Modulus in Elasticity (MPa)				0.2% Offset Yield Strength (MPa)			
Material	PCL	PCL/D10	PCL/D20	PCL/D30	PCL	PCL/D10	PCL/D20	PCL/D30
Mean	424	440	469	532	13.70	11.08	9.67	8.73
SD	2.94	1.41	5.35	5.35	0.44	0.25	0.41	0.22

**Table 4:** Mass loss value and standard deviation of PCL and composites for 9 weeks.

Weeks	PCL		PCL/D10		PCL/D20		PCL/D30	
	Mean	SD	Mean	SD	Mean	SD	Mean	SD
0	100		100		100		100	
3	99.60	0.07	93.40	0.02	84.19	1.43	74.79	0.60
6	99.09	0.01	93.23	0.06	82.67	0.23	72.91	0.10
9	98.38	0.17	92.71	0.26	82.33	0.45	70.60	0.21

**Table 5:** Water contact angle of various biomaterials.

Group		Materials	Water Contact Angle (°)	Refs.
Metal		Magnesium	40.8	[66]
		Tantalum	61	[67]
		Titanium	73	[68]
Ceramic		Alumina	64.74	[69]
		Zirconia	65	[70]
Polymer	Natural Polymer	Collagen	62.17	[71]
		Gelatin	78.6	[72]
		Chitosan	80	[73]
	Synthetic Polymer	PLA ^1^	87.2	[74]
		PCL	83.9	Current study
			118	[75]
		PGA ^2^	109.8	[76]
		PLGA ^3^	124.9	[64]

^1^ polylactic acid; ^2^ polyglycolide; ^3^ Polylactic-co-glycolic acid.

**Table 6:** Elastic modulus of PCL based composites.

Polymer Matrix	Additive	Additive Ratio (%)	Specimen	Test Method	Modulus (Mpa)	Refs.
PCL	-	-	Solid	Tensile test	440	[83]
	-	-	Solid	Three-point bending	414	[83]
	-	-	Solid	Compressive test	455	[83]
	-	-	Scaffold	Compressive test	10	[83]
	DMSO_2_	10	Molded bar	Three-point bending	440	Current study
	DMSO_2_	20	Molded bar	Three-point bending	469	Current study
	DMSO_2_	30	Molded bar	Three-point bending	532	Current study
	nHA ^1^	0	Cylindrical disk	Compressive test	71.72	[85]
	nHA	10	Cylindrical disk	Compressive test	67.65	[85]
	nHA	30	Cylindrical disk	Compressive test	68.55	[85]
	CNC ^2^	0	nano fiber	-	23.4	[49]
	CNC	0	nano fiber	-	33.1	[49]
	CNC	1	nano fiber	-	43.8	[49]
	CNC	1.5	nano fiber	-	39	[49]
	CNC	2.5	nano fiber	-	39.6	[49]
	CNC	4	nano fiber	-	27.8	[49]
	CNC	0		-	246.45	[86]
	CNC	5		-	205.95	[86]
	CNC	10		-	313.55	[86]
	CNC	15		-	460.5	[86]
	CNC	20		-	500.99	[86]
	CNC	25		-	629.42	[86]
	-	-	Scaffold	-	3.58	[87]
	MTA ^3^		Scaffold	-	4.07	[87]
	PCL grafted CNC	0	nano fiber	-	4.09	[88]
	PCL grafted CNC	1	nano fiber	-	4.49	[88]
	PCL grafted CNC	3	nano fiber	-	6.01	[88]
	PCL grafted CNC	5	nano fiber	-	6.94	[88]

^1^ nano hydroxyapatite; ^2^ cellulose nanocrystals; ^3^ mineral trioxide aggregate.

## Data Availability

The data presented in this study are available on request from the corresponding author.
